# Experience and perspectives of end-of-life care discussion and physician orders for life-sustaining treatment of Korea (POLST-K): a cross-sectional study

**DOI:** 10.1186/s12910-023-00897-x

**Published:** 2023-03-07

**Authors:** Hyeon-Su Im, Insook Lee, Shinmi Kim, Jong Soo Lee, Ju-Hee Kim, Jae Young Moon, Byung Kyu Park, Kyung Hee Lee, Myung Ah Lee, Sanghoon Han, Yoonki Hong, Hyeyeoung Kim, Jaekyung Cheon, Su-Jin Koh

**Affiliations:** 1 Division of Hematology and Oncology, Department of Internal Medicine, Ulsan University Hospital, Ulsan University College of Medicine, 877, Bangeojinsunhwando-ro, Dong-gu, Ulsan, 44033 Republic of Korea; 2 Department of Nursing, Changwon National University, 20 Changwon daehak-ro, Uichang-gu, Changwon, 51140 Republic of Korea; 3 Division of Nephrology, Department of Internal Medicine, Ulsan University Hospital, Ulsan University College of Medicine, Ulsan, Korea; 4 Department of Internal Medicine, Chungnam National University Sejong Hospital, Chungnam National University College of Medicine, Daejeon, Korea; 5 Division of Gastroenterology, Department of Internal Medicine, National Health Insurance Service Ilsan Hospital, Goyang, Korea; 6 Department of Internal Medicine, Yeungnam University College of Medicine, Daegu, Korea; 7 Division of Medical Oncology, Department of Internal Medicine, Seoul St. Mary’s Hospital, The Catholic University of Korea, Seoul, Korea; 8 Department of Hematology and Oncology, Jeju National University Hospital, Jeju National University College of Medicine, Jeju, Korea; 9 Department of Internal Medicine, Kangwon National University Hospital, Kangwon National University College of Medicine, Chuncheon, Korea; 10 Department of Medical Oncology, CHA Bundang Medical Center, CHA University School of Medicine, Seongnam, Korea

**Keywords:** End of life, Life-sustaining treatment, Healthcare provider

## Abstract

**Background:**

This study aimed to identify the healthcare providers’ experience and perspectives toward end-of-life care decisions focusing on end-of-life discussion and physician’s order of life-sustaining treatment documentation in Korea which are major parts of the Life-Sustaining Treatment Act.

**Methods:**

A cross-sectional survey was conducted using a questionnaire developed by the authors. A total of 474 subjects—94 attending physicians, 87 resident physicians, and 293 nurses—participated in the survey, and the data analysis was performed in terms of frequency, percentage, mean and standard deviation using the SPSS 24.0 program.

**Results:**

Study results showed that respondents were aware of terminal illness and physician’s order of life-sustaining treatment in Korea well enough except for some details. Physicians reported uncertainty in terminal state diagnosis and disease trajectory as the most challenging. Study participants regarded factors (related to relationships and communications) on the healthcare providers’ side as the major impediment to end-of-life discussion. Study respondents suggested that simplification of the process and more staff are required to facilitate end-of-life discussion and documentation.

**Conclusion:**

Based on the study results, adequate education and training for better end-of-life discussion are required for future practice. Also, a simple and clear procedure for completing a physician’s order of life-sustaining treatment in Korea should be prepared and legal and ethical advice would be required. Since the enactment of the Life-Sustaining Treatment Act, several revisions already have been made including disease categories, thus continuous education to update and support clinicians is also called for.

**Supplementary Information:**

The online version contains supplementary material available at 10.1186/s12910-023-00897-x.

## Background

With the recent development of novel and innovative medical treatments, life expectancy has been extended and withholding and/or withdrawal of any medical treatment is sometimes considered as failure in Korea [1]. This has caused an increase in aggressive treatment of terminally ill patients [2, 3]. In addition, as in the Boramae Hospital case (a representative case in which discussions on dying with dignity were raised in Korean society in 1997), the Supreme Court applied the physicians with aiding and abetting in a murder if a patient died after being discharged from hospital against medical advice [4]. The aforementioned have led to the some degree the excessive use of life-sustaining treatment (LST) by healthcare providers in Korea for the terminally ill or end-of-life (EOL) patients.

For medical care of terminally ill patients, various decisions are required and discussions about death and dying are inevitable, which used to be regarded as a social and medical taboo in Korea. However, people are aware that death is unavoidable, and they wish to have a meaningful life untill the end and to maintain quality of life even in the EOL period [5]. For EOL care decisions, autonomy manifested by self-determination reflecting people’s values and wishes is critical and people need to talk about death and dying in advance. In the process of EOL discussion, terminally ill patients and relevant stakeholders, usually healthcare providers and family members, share information and thoughts that could lead to an appropriate decision. Nevertheless, until recently, only a small number of patients have been directly involved in EOL discussions [6], despite some studies showing that most patients want to be involved in EOL discussions [7].

As of February 2018, the Act on Hospice and Palliative Care and Decisions on Life-sustaining Treatment for Patients at EOL (the “LST Act”), which advocates for patient autonomy and self-determination, has been enforced [8]. According to this law, a discussion on the end of life, which is usually recommended to be done in advance of end stage of life, is required. This discussion process is called advance care planning (ACP) and legal documentation including advance directives (AD) and physician’s orders for life-sustaining treatment (POLST) is completed as a result of this discussion. However, EOL discussion itself and relevant policies such as AD and POLST are new to healthcare providers in Korea. For that reason, the Ministry of Health and Welfare initiated a pilot project prior to full-scale implementation of LST Act. This paper reported the outcome of that pilot project. This pilot study was conducted focusing on two areas which would support more efficient law enforcement, based on the data drawn from the pilot project: EOL discussion and ACP documentation, especially completion procedures of POLST of Korea (POLST-K).

## Methods

### Aims

This study was performed to identify healthcare providers’ experiences and perspectives including awareness and opinions related to EOL discussion and POLST-K.

### Study design and settings

This cross-sectional study was conducted from October 2017 to January 2018 in seven hospitals, including teaching hospitals and general hospitals that participated in the pilot project promoted by the government across the nation.

### Participants

Study participants were physicians and nurses recruited from seven hospitals that agreed to participate in this study. Among the 200 physicians and 300 nurses who agreed to participate, 181 physicians’ and 293 nurses’ surveys were collected (response rate: 90.5% in physicians; 97.7% in nurses). A questionnaire on the experience and perspectives related to EOL care and discussion and POLST-K were administered to potential study respondents who were practicing or witnessing the EOL decision-making process during the pilot project period.

### Questionnaires and data collection

The questionnaire to solicit healthcare providers’ experience and perspectives was developed by the authors through rigorous literature and law analysis and validated by expert consultation. The consultation panel consisted of oncologists, hospice/palliative medicine specialists, internal medicine specialists and clinical nurses. In addition, a pilot study was also carried out with five physicians and five nurses to further refine items of the questionnaire. The final version of the questionnaire consisted of three categories, i.e., (1) participant characteristics, (2) experience and (3) perspectives. For experience, items relating to giving bad news and POLST-K completion were included (3 items). Under the perspective category, five subcategories were included as follows; (1) awareness of terminal illness care and POLST-K (11 items), (2) opinions about the factors that hinder terminal state diagnosis and giving bad news (13 items), (3) opinions about impediments to EOL discussion per stakeholder (13 items), (4) impediments to completion of POLST-K (6 items), and (5) suggestions and/or recommendations for future EOL care decision-making (10 items) respondents (Additional file 1). Due to the differences in the role of physicians and nurses, items on disease diagnosis and POLST were omitted from the survey of nurse respondents (Additional file 2).

The study was approved by the institutional review board (IRB) of Ulsan University Medical Center where the authors were based. The researchers explained the study purpose to the participants, and all data were collected after respondent’s permission was confirmed utilizing a written informed consent form. All participating healthcare providers completed the questionnaire anonymously and voluntarily. The study was carried out in accordance with the 1995 Helsinki Declaration and the ethical standards of National Research Committee. Data collected were handled confidentially in an approved manner.

### Statistical analysis

Data were analyzed using SPSS version 25.0 for Windows (SPSS Inc., Chicago, IL, USA). Descriptive statistics were used to explore healthcare providers’ experiences and perspectives of the EOL decision-making process according to the study aim.

## Results

### Participant characteristics

A total of 474 respondents (181 physicians and 293 nurses) participated in this study (Table 1). Of the physicians, 94 were attending physicians, and 87 were first to fourth-year resident physicians. The characteristics of the participants are shown in Table 1. A total of 26.7% of the physicians and 11.7% of the nurses had at least ten years clinical. A total of 58.6% of the physicians were in internal medicine, and 41.4% were from other departments including surgery, radiology, urology, ophthalmology, otolaryngology, etc. The primary disease reported by study participants in relation to patients for whom they had been caring was cancer (64.6% of the physicians and 83.6% of the nurses).

**Table 1:** Characteristics of the participants

Variable	n (%)^†^/Mean ± SD			
	Physicians			Nurses (n = 293)
	Total (n = 181)	Attending (n = 94)	Resident (n = 87)	
Age (years)	36.62 ± 8.11	42.40 ± 6.68	30.37 ± 3.64	33.53 ± 8.86
Gender				
Male	118 (65.2)	69 (73.4)	49 (56.3)	9 (3.1)
Female	63 (34.8)	25 (26.6)	38 (43.7)	284 (96.9)
Work experience (years)	6.57 ± 5.84	10.36 ± 5.92	2.54 ± 1.23	10.60 ± 9.07
≤ 1	15 (8.3)	7 (7.5)	8 (9.2)	48 (16.5)
1–5	93 (51.7)	16 (17.2)	77 (88.5)	107 (36.8)
5–10	24 (13.3)	22 (23.7)	2 (2.3)	102 (35.1)
≥ 10	48 (26.7)	48 (51.6)	–	34 (11.7)
Department				
Internal medicine	106 (58.6)	42 (44.7)	64 (73.6)	–
Non-internal medicine	75 (41.4)	52 (55.3)	23 (26.4)	–
Primary disease caring in practice				
Cancer	117 (64.6)	55 (58.5)	62 (71.3)	245 (83.6)
Non-cancer	64 (35.4)	39 (41.5)	25 (28.7)	48 (16.4)

*EoL* End-of-life, *AD* Advanced directives, *POLST* Physician orders for life-sustaining treatment

^†^Missing data excluded

### Experience of terminal illness and EOL care

**Fig. 1:**
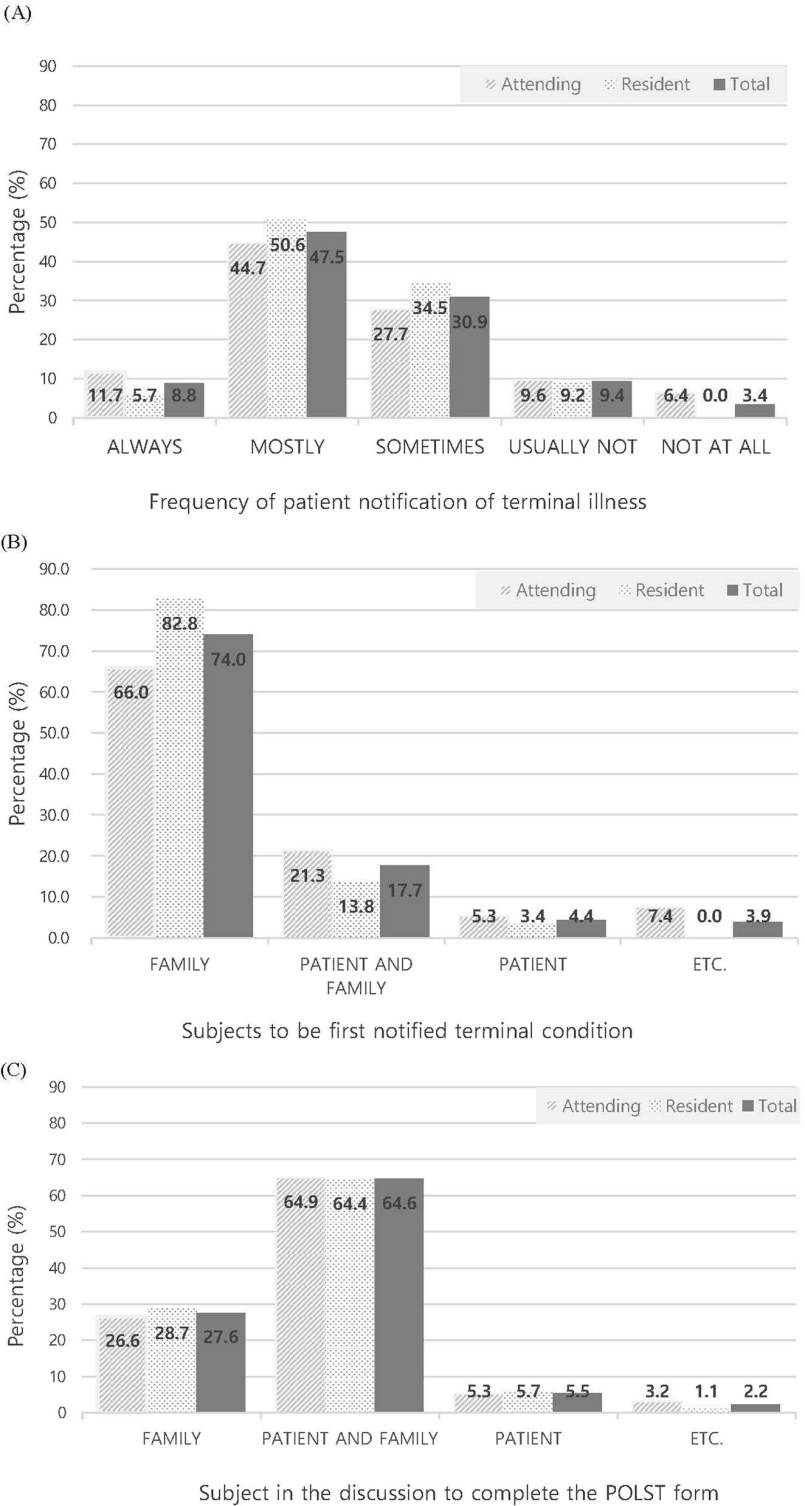
Experience in regarding terminal illness and EOL care. **A** Frequency of patient notification of terminal illness. **B** Subjects to be first notified of terminal illness. **C** Subjects in the discussion to complete the POLST form

Experience of terminal and EOL care are presented in Table 2 and Fig. 1. About half of the physicians had made a terminal diagnosis in more than two cases per month and 80% of physicians and 70% of nurses reported that they had some experience with POLST-K. Overall satisfaction level about EOL decisions appeared evenly (about 50%) across the subgroups.

**Table 2:** Experience of the terminal and EOL care

Variable	n (%)^†^/Mean ± SD			
	Physicians			Nurses (n = 293)
	Total (n = 181)	Attending (n = 94)	Resident (n = 87)	
Number of terminal diseases diagnosed per month (range: 0–20)				
≤ 2.0	85 (47.5)	59 (62.8)	42 (49.4)	–
> 2.0	94 (52.5)	35 (37.2)	43 (50.6)	–
Acquaintance with POLST-K				
Yes	145 (80.1)	79 (84.0)	66 (75.9)	205 (70.0)
No	36 (19.9)	15 (16.0)	21 (24.1)	88 (30.0)
Satisfaction with EOL decisions (range: 0–100)	51.47 ± 20.63	50.42 ± 19.65	51.10 ± 20.20	56.86 ± 17.99

POLST-K Physician orders for life-sustaining treatment-Korean

^†^Missing data excluded

Fifty-six percent of the physician respondents reported that they either always or mostly talked to patients about terminal state diagnosis (Fig. 1A). Seventy-four percent of the physicians notified the family members first about the patients’ terminal state, and 22.1% notified both the patient and family members at the same time (Fig. 1B). Physicians completed the POLST-K form along with a discussion with patients and family members (64.6%), family members only (27.6%), and patient only (5.5%), respectively. The study found that 29.9% of patients were excluded from the EOL decision-making process according to the physicians’ report (Fig. 1C).

### Perspectives on EOL discussion

#### Awareness of terminal illness and POLST-K

Regarding terminal illness, 50.3% of the physicians and 83.8% of the nurses answered that stage 4 cancer meant terminal illness which is not all ways true. Approximately 30% of the physicians and nurses considered that terminal illnesses can be cured after adequate medical treatment. However, 85.6% of the physicians and 79.5% of the nurses answered that “the survival rate of patients with advanced cancer after cardiopulmonary resuscitation is usually less than 10%.” Almost 95.0% of respondents reported that patients should be notified of a terminal illness. While 18.2% of the physicians and 16.7% of the nurses said that hospice palliative care would mean hopelessness to patients with a terminal illness, 86.7% of the physicians and 97.3% of the nurses considered that hospice palliative care could be more helpful than aggressive treatment (Table 3).

**Table 3:** Awareness of terminal illness and POLST-K

Contents	n (%)^†^			
	Physicians			Nurses (n = 293)
	Total (n = 181)	Attending (n = 94)	Resident (n = 87)	
Stage 4 cancer is a terminal illness	90 (49.7)	55 (58.5)	35 (40.2)	47 (16.2)
Terminal illness can be cured with adequate medical treatment	125 (69.1)	67 (71.3)	58 (66.7)	201 (68.6)
The survival rate of patients with advanced cancer after CPR is usually less than 10%	155 (85.6)	79 (84.0)	76 (87.4)	233 (79.5)
Notifying patients of terminal illness is commendable	172 (95.0)	91 (96.8)	81 (93.1)	277 (94.9)
Hospice palliative care is recommendable for patients with a terminal disease	157 (86.7)	80 (85.1)	77 (88.5)	285 (97.3)
Hospice palliative care would mean hopelessness to patients with a terminal disease	148 (81.8)	80 (85.1)	68 (78.2)	244 (83.3)
CPR, mechanical ventilation, hemodialysis, chemotherapy is LST option included in POLST-K	144 (79.6)	80 (85.1)	64 (73.6)	234 (79.9)
POLST-K should be introduced and explained by a physician before it is completed	96 (53.0)	55 (58.5)	41 (47.1)	191 (65.2)
POLST-K cannot be changed or abolished after completion	162 (89.5)	85 (90.4)	77 (88.5)	259 (88.4)
POLST-K can be completed based on the decision of family members	84 (46.4)	47 (50.0)	37 (42.5)	137 (46.8)
POLST-K can be displaced by DNR	131 (72.4)	71 (75.5)	60 (69.0)	168 (57.3)

*EOL* End-of-life; *POLST-K* Physician’s order of life-sustaining treatment; *CPR* Cardiopulmonary resuscitation; *LST* Life-sustaining treatment

^†^Correct response, missing data excluded

In relation to the contents of POLST-K, the level of awareness about the decision-maker was very low across all respondents, while physicians and nurses all revealed high awareness relating to document updating and/or abolition. Also, the majority of physicians and nurses considered that do not resuscitate (DNR) could replace POLST-K in the future.

#### Physicians’ opinions on impediments to terminal state diagnosis and giving bad news

Difficulties regarding terminal illness diagnosis and giving bad news to patients are shown in Table 4. Physicians reported that the uncertainty of the disease trajectory (34.1%) and ambiguity of the diagnosis criteria (30.1%) hindered timely diagnosis of a terminal illness. Worries about the patients’ frustration or disappointment (27.9%) and family members’ objection to telling the truth to patients (25.3%) were the main factors that prohibited telling patients about their terminal illness.

**Table 4:** Opinions on impediment of terminal state diagnosis and speaking bad news

	n (%)^†^		
	Total (n = 181)	Attending (n = 94)	Resident (n = 87)
*Terminal state diagnosis*			
Uncertainty of disease course	119 (34.1)	66 (36.3)	53 (31.7)
Ambiguity of terminal state diagnosis criteria	105 (30.1)	51 (28.0)	54 (32.3)
Discomfort with legal responsibility	58 (16.6)	29 (15.9)	29 (17.4)
Unawareness of the terminal state diagnosis	37 (10.6)	12 (6.6)	25 (15.0)
Consideration of terminal diagnosis as unnecessary due to the possibility of giving up on treatment	15 (4.3)	11 (6.0)	4 (2.4)
Others	15 (4.3)	13 (7.1)	2 (1.2)
Total	349 (100.0)	182 (100.0)	167 (100.0)
*Speaking bad news*			
Patient’s disappointment and frustration	100 (27.9)	54 (29.2)	46 (26.4)
Family members’ opposition	91 (25.3)	46 (24.9)	45 (25.9)
Fear that the patient would think the worst case	55 (15.3)	28 (15.1)	27 (15.5)
Feeling of giving up	41 (11.4)	16 (8.6)	25 (14.4)
Time constraint	25 (7.0)	13 (7.0)	12 (6.9)
Patient’s request for maintenance of treatment	19 (5.3)	10 (5.4)	9 (5.2)
Patient’s incomprehension of the condition	9 (2.5)	7 (3.8)	2 ( 1.1)
The risk of breaking rapport	9 (2.5)	4 (2.2)	5 (2.9)
Others	10 (2.8)	7 (3.8)	3 (1.7)
Total	359 (100.0)	185 (100.0)	174 (100.0)

^†^Multiple responses (two of the most significant items were selected), missing data excluded

From a total of 362 responses relating to impediments to EOL discussions with patients or family members, factors on the physicians’ side were considered the main impediments (75.7%) (Table 5). The top most factor was uncertainty about the appropriate timing of the EOL discussion (26.0%). Family members’ negative attitudes and disagreement among family members about EOL discussion were recognised as major barriers among 13% of physician respondents, while 11.3% of physicians considered patients’ factors as the key barriers to EOL discussion. The main impediment to completing the POLST-K document was the complicated procedures (20.6%) (Table 6).

**Table 5:** Opinions on impediment to EOL discussion by stakeholder

Categories	Items	n (%)^†^		
		Total (n = 181)	Attending (n = 94)	Resident (n = 87)
Physicians	Uncertainty of the timing	94 (26.0)	50 (26.6)	44 (25.3)
	Concerns of patient’s disappointment and/or frustration	76 (21.0)	40 (21.3)	36 (20.7)
	Thought of giving up on patients	53 (14.6)	22 (11.7)	31 (17.8)
	Inability to explain the situation	26 (7.2)	13 (6.9)	13 (7.5)
	Time constraint	25 (6.9)	10 (5.3)	15 (8.6)
	Subtotal	274 (75.7)	135 (71.8)	139 (79.9)
Patients	Patients’ incomprehension on the purpose of LST	14 (3.9)	8 (4.3)	6 (3.4)
	Patients’ wish for physicians to decide on their behalf	11 (3.0)	7 (3.7)	4 (2.3)
	Patients’ reluctance	9 (2.5)	5 (2.7)	4 (2.3)
	Patients’ incomprehension of the relevant terms	5 (1.4)	3 (1.6)	2 (1.1)
	Patients’ wish for family members to decide on their behalf	2 (0.6)	1 (0.5)	1 (0.6)
	Subtotal	41 (11.3)	24 (12.8)	17 (9.8)
Family members	Family members’ opposition against open discussion	20 (5.5)	10 (5.3)	10 (5.7)
	Disagreement among family members	20 (5.5)	13 (6.9)	7 (4.0)
	Family members making decisions for patients	4 (1.1)	4 (2.1)	0 (0.0)
	Others	3 (0.8)	2 (1.1)	1 (0.6)
	Subtotal	47 (13.0)	29 (15.4)	18 (10.3)
Total		362 (100.0)	188 (100.0)	174 (100.0)

*EOL* End-of-life; *LST* Life-sustaining treatment

^†^Multiple responses (two of the most significant items were selected), missing data excluded

**Table 6:** Impediment to POLST-K completion of physicians

Categories	n (%)^†^		
	Total (n = 181)	Attending (n = 94)	Resident (n = 87)
Complicated procedures	74 (20.6)	38 (20.4)	36 (20.8)
Difficulty in obtaining a consensus of family members	68 (18.9)	35 (18.8)	33 (19.1)
Patient’s lack of self-determination	65 (18.1)	32 (17.2)	33 (19.1)
Too many documents to go through	58 (16.2)	21 (11.3)	37 (21.4)
Legal requirement for terminal state diagnosis	21 (5.8)	15 (8.1)	6 (3.5)
Disagreement among the family members	14 (3.9)	9 (4.8)	5 (2.9)
Total	359 (100.0)	186 (100.0)	173 (100.0)

*EOL* End-of-life

^†^Multiple responses (two of the most significant items were selected), missing data excluded

#### Suggestion for future EOL discussion and POLST-K

**Fig. 2:**
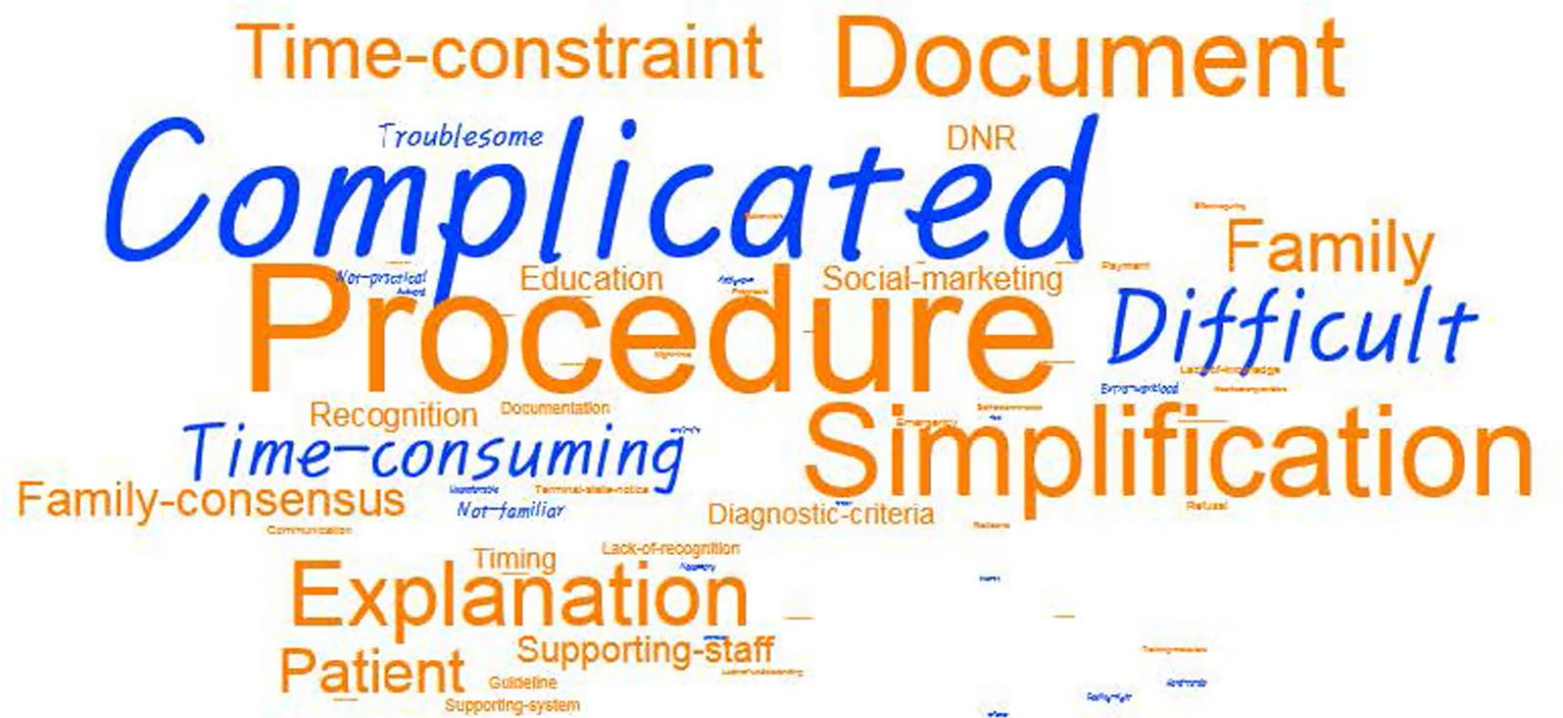
Key words expressed by physicians related by POLST-K documentation

From a list of 10 potential actions to increase EOL discussion and POLST-K completion, over 40% of physicians, considered that simplifying the POLST-K completion process was the highest priority to facilitate EOL discussion and POLST-K documentation, while nurses reported that sufficient staffing would be the biggest barrier. (Table 7). Keywords expressed by physicians related to POLST-K completion and its utility in future are outlined in Fig. 2.

**Table 7:** Suggestion for future EOL discussion and POLST-K completion

Categories	n (%)^†^			
	Physicians			Nurses (n = 293)
	Total (n = 181)	Attending (n = 94)	Resident (n = 87)	
Simplification of the procedures	74 (40.9)	37 (39.4)	37 (42.5)	82 (28.0)
Grant physicians immunity against criminal, civil, or disciplinary sanctions	51 (28.2)	30 (31.9)	21 (24.1)	54 (18.4)
Sufficient human resources	49 (27.1)	21 (22.3)	28 (32.2)	118 (40.3)
Provision of tools for predicting the disease course	47 (26.0)	23 (24.5)	24 (27.6)	63 (21.5)
Standardized guidelines for POLST-K completion	38 (21.0)	21 (22.3)	17 (19.5)	73 (24.9)
Liaison services for ethical and legal consultation	37 (20.4)	24 (25.5)	13 (14.9)	40 (13.7)
Adequate reward for the process itself	20 (11.0)	13 (13.8)	7 (8.0)	11 (3.8)
Education for better communication	19 (10.5)	9 (9.6)	10 (11.5)	67 (22.9)
Provision of patient education materials, such as video clips or leaflets	17 (9.4)	8 (8.5)	9 (10.3)	54 (18.4)
Applying in conjunction with DNR	9 (5.0)	2 (2.1)	7 (8.0)	15 (5.1)

*EOL* End-of-life; *POLST* Physician orders for life-sustaining treatment; *DNR* Do not resuscitate

^†^Multiple responses (two of the most significant items were selected)

## Discussion

It has been years since the LST Act was enacted in 2018, and it is reported that the POLST-K completion rate is continuously increasing and data related to this Act are being presented [9, 10]. However, research conducted on EOL discussion and POLST-K in direct connection with this law is still lacking. Thus we have attempted to present relevant data and opinions for better practice in the future from healthcare providers’ point of view.

In this study, 74.0% of physicians informed the patient’s family about a terminal diagnosis before telling the patient. In EOL discussions, including giving bad news and providing LST options, the social and emotional environments are key factors [11]. Korea is well known to be a family-oriented society, and family members are the main and preferential stakeholders in a conversation about EOL care planning [12] which often leads to patients’ autonomy in the decision-making process on EOL care being overlooked. Nevertheless, the LST Act clearly states that the patient is the one who should participate in the discussion about EOL care decisions, knowing his/her disease state and making their own decisions. Therefore, according to the LST Act, physicians should tell the patient about EOL care options as well as a disease state. However, because of a long-standing cultural tradition of telling families first, some physicians would not find that easy to do; this might explain why 74.0% of physicians told bad news to patients’ families first. Therefore, various communication strategies including education and guidelines are being proposed. Likewise, strategies that reflect Korean culture, law, and clinical environment will be needed to facilitate EOL discussions.

The majority of physicians and nurses appeared to be aware of what is classified as terminal illness, albeit with relatively low levels of awareness in some aspects. To be more specific, most nurses reported that they considered stage 4 cancer as a terminal stage but this is not always the case. This is a surprising finding in that healthcare providers' attitudes toward disease can influence patient care and needs to be corrected.

The majority of study respondents reported that they were familiar with the important contents of POLST-K. However, the level of awareness of the POLST-K completion process and decision-makers was not satisfactory. Since this study is an analysis of data from the pilot project in 2017, we expected that the level of awareness would have increased since the time of data collection. On the contrary, it is reported that the overall awareness of resident physicians has not changed over time even though a majority of resident physicians responded that they were educated about the LST Act [13]. Therefore, more practical strategies are required in terms of education technology and content. For example, education in the clinical setting makes a significant difference to overall awareness about the LST Act [13], and on-site education might be one important option to consider. Education programs are especially helpful when awareness or knowledge issues are more concerned in EOL discussion [14]. In addition to awareness issues, communication about EOL decisions is well known to be challenging to clinicians and simple solutions are unlikely to be effective [15]. Therefore, education or training programs including adequate content and ‘how to’ issues would be necessary.

Physician respondents reported that most of their difficulties in diagnosing terminal illnesses were due to uncertainty of the disease trajectory and the ambiguity of the terminal state’s diagnostic criteria. This result is supported by a systematic review [16] which reported that prognostic uncertainty was a key factor to hinder EOL discussion. For this reason, opinions on terminal state diagnosis vary among physicians [17] and the Korean Academy of Medical Sciences has published consensus guidelines [18]. However, application of the guidelines in actual practice seems still challenging, judging from the results of this study. In fact, disease trajectory itself implies uncertainty, which also needs to be acknowledged. Thus, various aspects such as a patient’s functional status, signs and symptoms, and available laboratory data also should be considered along with disease state [19]. In addition, there are various tools for diagnosing terminal diseases that were constructed using the aforementioned factors. Examples of these tools include the Palliative Prognostic Score [20], Palliative Prognostic Index [21], or Surprise question [22]. These tools were developed more than a decade ago and have been widely promoted; implementation of these tools in clinical practice would help to overcome the ambiguity of diagnosing terminal illnesses.

When the discussion is actually initiated, ACP document completion rate is found to increas [23]. In addition to awareness or knowledge-related factors, each patient's attitude toward EOL care that is influenced by their beliefs and cultural background is important [11] and each patient’s wishes and needs also should be taken into account in the discussion. In addition, the conversation guide for serious illness recommends exploring the patient’s values and discussing goals of care and preferences for life-sustaining interventions before completing the POLST documents [24].

Even after a terminal state is confirmed, informing patients of their condition and initiating discussions on EOL care is a still difficult issue for healthcare providers. Nonetheless, guidelines suggest that a conversation about EOL care decisions should be maintained even after the diagnosis of life-threatening disease in clinical practice [25]. Impediments to informing patients about their terminal state could hinder timely initiation of the EOL discussion and specific barriers were revealed in this study. The top two factors for physicians to be reluctant to give bad news to patients were fear of the patient’s disappointment/frustration and family members’ opposition. However, the LST Act clearly specifys having EOL discussions with patients and ACP documentation rate is found to be increased when the discussion is actually initiated [23]. ACP documents, especially POLST are made through a structured discussion and allow integration of the patient’s values and the physician’s expertise [26] which can promote the patient’s best interest. In addition, when it comes to communication issues, barriers to hinder effective conversation could be overcome with increasing confidence with EOL care conversations by communication skills [27], which also can be improved with appropriate strategies [15].

We identified factors hindering EOL discussions by stakeholder groups of physicians, patients, and families, and most of the factors reported as impediments to EOL discussions turned out to be physician-side factors. Among those, the uncertainty of appropriate EOL discussion timing, concerns about the patient’s disappointment/frustration, and the thought of giving up on the patient’s life were the main barriers reported by the physicians. These impediments are mainly procedure-related and could be solved by education and training including communication skills. Physicians’ education on EOL care was reported to be insufficient, which led to a lack of confidence during EOL discussions with patients and caregivers [28]. When appropriate EOL communication skill training is provided, it can improve EOL discussion and increase the ACP document completion rate as well [29]. Therefore, EOL discussions need to be part of routine medical care for patients with a terminal disease. The Royal College of Physicians in the United Kingdom released the “Talking About Dying” report in 2018, which contains practical advice to physicians on EOL conversations [30]. This type of training and learning materials with clearly defined objectives, a structured curriculum, evaluation methods, and feedback mechanisms would be helpful.

Patient- or family-related factors were much less an issue in end-of-life communication than physician-related factors. Nevertheless, effort to resolve a small portion of these problems can make big difference and ACP, along with shared decision-making (SDM) through family meetings or embedding EOL discussion into everyday practice, would be helpful for patients facing death.

Factors perceived as impediments and suggestions to overcome them in completing POLST-K were consistent as shown in Fig. 2 and Table 6, and they were mainly procedure-related. A domestic study evaluating the feasibility of the POLST-K discussion for patients with terminal cancer reported that only about one-third of the patients had a POLST form completed [31]. Fortunately, recent statistics [9] show that feasibility is improving over time as experience accumulates; however more effort still is needed to facilitate POLST-K preparation through simple and clear procedures.

Based on the results of this study, it is important to elicit the opinions of patients and their families as well to obtain their agreement. In a clinical setting, disagreement among stakeholders including patients, family members and healthcare providers is not unusual. Especially in relation to disagreements between patients and family members, a qualitative study on LST decision-making in Korea proposed ‘a context-oriented model for EOL communication’ [32]. This study suggested that healthcare providers need to identify the decision-making dynamics between the patient and family first, and then determine the patient’s willingness to make decisions independently or jointly with family members (or at least those family members who are willing to participate in decision-making). When the patient or family members accept the terminal state and are ready to participate in the decision-making process, effective EOL discussion and adequate decision-making are more likely [32].

## Conclusion

This study explored practical details and suggestions in applying the LST Act focusing on healthcare providers’ experience and perspectives toward EOL discussion. The study results are derived from clinicians directly involved in practice related to the LST Act, thus having important implications for present and future EOL decisions especially related to the LST Act. In conclusion, this study revealed factors that impede the diagnosis of terminal illnesses and EOL discussion. Physician-related factors were the main impediment to the EOL discussion and to overcome these barriers, approaches including on-site education are required. In addition, patient and family knowledge and awareness of related issues must also be improved through appropriate strategies such as social marketing.

This study also had several limitations. First, this study involved only healthcare providers, not patients and their family members. To accurately determine impediments, studies involving patients and family members need to be conducted. Second, this study was conducted just before the implementation of the LST Act, therefore further studies exploring the influence of this Act on EOL discussion are called for.

## Supplementary Information

**Additional file 1.** Life-sustaining treatment pilot project questionnaire for doctors.

**Additional file 2.** Life-sustaining treatment pilot project questionnaire for nurses.

## Data Availability

All data generated and analyzed during this study are included in this published article. Data are available from the corresponding author on reasonable request.

## Abbreviations

ACP: Advance care planning
AD: Advance directives
DNR: Do not resuscitate
EOL: End-of-life
IRB: Institutional review board
LST: Life-sustaining treatment
POLST: Physician’s orders for life-sustaining treatment
POLST-K: Physician’s orders for life-sustaining treatment of Korea
SDM: Shared decision-making
