# Supplementary material for: Experience and perspectives of end-of-life care discussion and physician orders for life-sustaining treatment of Korea (POLST-K): a cross-sectional study
Source: BMC Med Ethics. 2023 Mar 7;24:18. doi: 10.1186/s12910-023-00897-x (PMC9993746; doi:10.1186/s12910-023-00897-x)
Supplement: Supplementary file 1 — Additional file 1. Life-sustaining treatment pilot project questionnaire for doctors. [file 12910_2023_897_MOESM1_ESM.docx]

**Additional file 1.** Life-sustaining treatment pilot project questionnaire for doctors

**General characteristics**

1. What is your sex? 1) Male 2) Female

2. How old are you? ( ) years old in the international age

3. What is your religion? 1) Christianity 2) Buddhism 3) Catholicism 4) None 5) Other ( )

4. How long is your work experience? ( ) years ( ) months

5. What is your current position at the hospital or institution at which you work?

1) Intern 2) Resident 3) Fellow

6. What is your department?

1) Department of Family Medicine 2) Internal Medicine (sub-specialty: )

3) Department of Anesthesiology and Pain Medicine 4) Department of Radiation Oncology

5) Pathology 6) Urology 7) Obstetrics and Gynecology 8) Plastic Surgery 9) Pediatrics

10) Neurology 11) Neurosurgery 12) Ophthalmology 13) Radiology 14) Surgery

15) Department of Emergency Medicine 16) Department of Biomedical Engineering

17) Department of Otolaryngology 18) Department of Clinical Pharmacology

19) Department of Rehabilitation Medicine 20) Department of Psychiatric Medicine

21) Orthopedic Surgery 22) Department of Diagnostic and Laboratory Medicine

23) Dermatology 24) Department of Nuclear Medicine 25) Cardiothoracic Surgery

26) Intern 27) Department of Occupational Medicine

**I. Experience in end-of-life care**

7. What are the primary medical conditions that you treat? (Multiple responses)

1) Cancer 2) AIDS 3) COPD 4) LC 5) Chronic heart disease 6) Cerebrovascular disease 7) Kidney disease 8) Neurodegenerative disease 9) Dementia 10) Other ( )

8. How often do you diagnose terminally ill patients? ( ) times/month

9. How satisfied are you with the treatment decisions for terminally ill patients?

|  |  |  |  |  |  |  |  |  |  |
| --- | --- | --- | --- | --- | --- | --- | --- | --- | --- |
|  |  |  |  |  |  |  |  |  |  |

0% 20 40 60 80 100%

(Not satisfied at all) (Very satisfied)

**II. Diagnosing terminal stages**

10. What are the two most difficult ***factors*** when diagnosing a terminal stage? 1st ( ), 2nd ( )

1. The possibility of curing a disease should not be given up; thus, there is no need to make a terminal diagnosis.
2. The terminal diagnosis is difficult due to the uncertain prognosis of the patient.
3. I do not know the diagnostic criteria for the terminal stage.
4. The criteria for terminal diagnosis are ambiguous.
5. Being legally responsible is burdensome.
6. Other ( )

**III. Notifying terminal stages**

11. **Who** is the first person that you inform of the terminal condition?

1) Patient 2) Family 3) Both patient and family 4) Other ( )

12. Do you directly inform the patient about the terminal condition?

1. Always
2. Mostly
3. Sometimes
4. Mostly no
5. Never

13. What are the ***two most difficult factors*** when directly communicating a terminal condition to a patient? 1^st^ ( ), 2^nd^ ( )

1. I feel that I am not doing my best as a doctor and that I am giving up on my patients.
2. It is difficult to explain the terminal stage due to lack of treatment hours.
3. Patients may get disappointed or frustrated and lose the will to live.
4. Patients feel anxious regarding their disease getting worse.
5. Relationships of trust with patients could get broken.
6. Patients demand to be treated till the end.
7. Patients do not understand the term “terminal.”
8. Family members are against informing the patient of the terminal illness.
9. Other ( )

14. Have you heard of “advance directives?” 1) Yes 2) No

15. Have you heard of “life-sustaining medical plan?” 1) Yes 2) No

16. Please place a checkmark for each content about “life-sustaining medical plan.”

| **Contents** | **No** | **yes** | **Do not know** |
| --- | --- | --- | --- |
| 1. A life-sustaining medical plan is a document that states what a relatively healthy patient wants or does not want to receive in case they lose their decision-making ability. |  |  |  |
| 1. In a life-sustaining medical plan, life-sustaining medical treatments refer to cardiopulmonary resuscitation, wearing a ventilator, hemodialysis, and administration of anticancer drugs. |  |  |  |
| 1. A life-sustaining medical plan can only be prepared when a doctor directly explains it to patients. |  |  |  |
| 1. Once a life-sustaining medical plan is written, it cannot be changed. |  |  |  |
| 1. A life-sustaining medical plan can be prepared by family members. |  |  |  |
| 1. You can use a DNR form instead of a life-sustaining medical plan. |  |  |  |
| 1. A life-sustaining medical plan includes options for using hospice palliative care. |  |  |  |

17. Please place a checkmark for each content related to end-of-life and terminal care.

| **Contents** | **Strongly agree** | **Agree** | **Disagree** | **Strongly disagree** |
| --- | --- | --- | --- | --- |
| 1. Stage 4 cancer refers to a terminal stage. |  |  |  |  |
| 1. It is better to inform the patient of their terminal condition. |  |  |  |  |
| 1. Hospice and palliative care can lend a sense of hopelessness to the patients. |  |  |  |  |
| 1. Hospice and palliative care are more helpful compared to active care for terminally ill patients. |  |  |  |  |
| 1. The chance of survival after CPR in patients with advanced cancer is less than 10%. |  |  |  |  |
| 1. Terminally ill patients can recover with proper treatments. |  |  |  |  |

**IV. Beginning of discussion about decisions on life-sustaining treatment**

18. When do you explain and discuss life-sustaining treatment decisions?

1. When the patient is healthy
2. When the patient is diagnosed with a disease
3. During treatment
4. When the condition gets worse
5. When the patients is diagnosed with the terminal stage
6. A few days before predicted death
7. Other ( )

**V. Discussion of life-sustaining treatment decisions – subjects, methods, etc.**

19. To whom do you explain and discuss life-sustaining treatment decisions?

1. Patient
2. Family
3. Both the patient and family
4. Other ( )

20. What are the two most challenging factors when discussing life-sustaining treatment decisions? 1^st^ ( ), 2^nd^ ( )

**(Doctor factor)**

1. While it has been predicted to be terminal, there is no certainty if the current condition is appropriate for life-sustaining treatment decisions.
2. I am reluctant to explain the discontinuation of life-sustaining medical care because I feel as if I am giving up.
3. Due to the lack of treatment hours, explaining a life-sustaining treatment is impossible.
4. I need to learn how to explain patients’ life-sustaining treatment decisions.
5. After explaining the discontinuation of life-sustaining medical care, I worry that patients are disappointed and frustrated.

**(Patient Factor)**

1. Patients need to understand the purpose and intent of the life-sustaining treatment decision.
2. Patients do not understand terms such as “cardiopulmonary resuscitation” and “ventilator.”
3. Patients are hesitant or burdened to make decisions about life-sustaining medical care.
4. Patients want their doctor to decide on life-sustaining medical care.
5. Patients want their families to decide on life-sustaining medical care.

**(Family factor)**

1. Family members are against explaining life-sustaining treatment decisions to the patients.
2. Family members want to make life-sustaining treatment decisions on behalf of the patients.
3. It is challenging to coordinate opinions due to conflicts or disagreements within the family.
4. Other ( )

21. What do you think are the two most important things needed ***to facilitate discussions about life-sustaining medical decisions***? 1^st^ ( ), 2^nd^ ( )

1. We need tools that can objectively assess the prognosis.
2. Documentation related to life-saving treatment decisions should be streamlined to reduce the time required for administrative procedures.
3. There needs to be compensation for the time spent discussing life-sustaining treatment decisions.
4. Training in communication should be provided to facilitate better discussion of life-sustaining treatment decisions.
5. Regulations should be placed to protect medical personnel when making life-sustaining treatment decisions.
6. There is a need for materials (leaflets, videos) to explain life-sustaining treatment decisions to patients.
7. There is a need for personnel who can consult with patients and families about life-sustaining treatment decisions.
8. There is a need for a team of experts who can provide ethical and legal advice on life-sustaining treatment decisions.
9. Standardized guidelines are needed for life-sustaining treatment decisions.
10. It is necessary to maintain the existing DNR.
11. Other ( )

**VI. Decision to discontinue the end-of-life treatment**

22. What do you think are the ***two most difficult decisions*** about the discontinuation of end-of-life treatment? 1^st^ ( ), 2^nd^ ( )

1. It is difficult for an attending physician and two fellows of related specialties to determine the end-of-life stage.
2. The process involved in decisions to discontinue end-of-life treatment is complex.
3. There are too many documents related to decisions to discontinue end-of-life treatment.
4. At the end-of-life stage, patients are unconscious, making it difficult to confirm their will to discontinue life-sustaining treatment.
5. Getting signatures, video records, and voice records for end-of-life patients is practically difficult.
6. In many cases, getting agreements from the entire family is impossible.
7. At the end of life, family opinions are more important.
8. Other ( )
