# Supplementary material for: Experience and perspectives of end-of-life care discussion and physician orders for life-sustaining treatment of Korea (POLST-K): a cross-sectional study
Source: BMC Med Ethics. 2023 Mar 7;24:18. doi: 10.1186/s12910-023-00897-x (PMC9993746; doi:10.1186/s12910-023-00897-x)
Supplement: Supplementary file 2 — Additional file 2. Life-sustaining treatment pilot project questionnaire for nurses. [file 12910_2023_897_MOESM2_ESM.docx]

**Additional file 2.** Life-sustaining treatment pilot project questionnaire for nurses

**General characteristics**

1. What is your sex? 1) Male 2) Female

2. How old are you? ( ) years old in the international age

3. What is your religion? 1) Christianity 2) Buddhism 3) Catholicism 4) None 5) Other ( )

4. How long is your work experience? ( ) years ( ) months

5. Are you currently providing care for a patient with a life-threatening condition who is expected to die within one year?

1) Yes, **☞ Go to Question 6** 2) No, **☞ Go to Question 7**

6. (If answered yes for Question 5) Select the patients who are expected to die within one year.

1) Adult cancer patients 2) Adult non-cancer patients 3) Children and adolescents 4) Other ( )

7. What are the primary terminal illnesses that you provide care for? (Multiple responses)

1) Cancer 2) AIDS 3) COPD 4) LC 5) Chronic heart disease

6) Cerebrovascular disease 7) Kidney disease 8) Neurodegenerative disease

9) Dementia 10) Other ( )

8. How often does the doctor you work with inform the patient of their terminal condition?

1. Always
2. Mostly
3. Sometimes
4. Mostly not
5. Never

9. How satisfied are you with the treatment decisions for terminally ill patients?

|  |  |  |  |  |  |  |  |  |  |
| --- | --- | --- | --- | --- | --- | --- | --- | --- | --- |
|  |  |  |  |  |  |  |  |  |  |

0% 20 40 60 80 100%

(Not satisfied at all) (Very satisfied)

10. Have you heard of “advance directives?” 1) Yes 2) No

11. Have you heard of “life-sustaining medical plan?” 1) Yes 2) No

12. Please place a checkmark for each content about “life-sustaining medical plan.”

| **Contents** | **No** | **Yes** | **Do not know** |
| --- | --- | --- | --- |
| 1. A life-sustaining medical plan can only be prepared when a doctor explains it directly to patients. |  |  |  |
| 1. A life-sustaining medical plan is a document that states the d decisions of a relatively healthy patient regarding what they wants or do not want to receive in case they lose their decision-making ability. |  |  |  |
| 1. In a life-sustaining medical plan, life-sustaining medical treatments refer to cardiopulmonary resuscitation, wearing a ventilator, hemodialysis, and administration of anticancer drugs. |  |  |  |
| 1. Once a life-sustaining medical plan is written, it cannot be changed. |  |  |  |
| 1. A life-sustaining medical plan can be prepared by family members. |  |  |  |
| 1. You can use a DNR form instead of a life-sustaining medical plan. |  |  |  |
| 1. A life-sustaining medical plan includes options for using hospice palliative care. |  |  |  |

13. Please place a checkmark for each content related to end-of-life and terminal care.

| **Contents** | **Strongly agree** | **Agree** | **Disagree** | **Strongly disagree** |
| --- | --- | --- | --- | --- |
| 1. Stage 4 cancer refers to a terminal stage. |  |  |  |  |
| 1. It is better to inform the patient of their terminal condition. |  |  |  |  |
| 1. Hospice and palliative care can lend a sense of hopelessness to patients. |  |  |  |  |
| 1. Hospice and palliative care are more helpful compared to active care for terminally ill patients. |  |  |  |  |
| 1. The chance of survival after CPR in patients with advanced cancer is less than 10%. |  |  |  |  |
| 1. Terminally ill patients can recover with proper treatments. |  |  |  |  |

14. What are the two most important reasons that patients are referred to hospice and palliative care? 1^st^ ( ), 2^nd^ ( )

1. Management of patients' physical symptoms
2. Psychological and spiritual care for patients
3. Consultation on advance care plan (life-sustaining treatment plan or discontinuation of life-sustaining care)
4. End-of-life counseling and care
5. Reference for inpatient or home hospice
6. Reference regarding community resources for patients and families (living support, care support, etc.)
7. Care for family members and counseling
8. Therapy programs for patients and family members
9. Care for those who have lost their family members
10. Other ( )

15. What do you think are the two things that make referrals for hospice and palliative care the most difficult? 1^st^ ( ), 2^nd^ ( )

1. Refusal by patients or family.
2. Difficulty in deciding when to refer for hospice or palliative care
3. Not familiar with the process of referring to hospice and palliative care
4. Concerned that patients and families consider it as “giving up on the patient”
5. Hospice and palliative care are considered as doing nothing
6. Hospice and palliative care are regarded as not significantly helpful to patients
7. No hospice referral is required as good care for patients can be provided till the end
8. Other ( )

16. Do you explain your life-sustaining treatment plan to your patients?

1) Yes **☞ Go to Question 17**

2) No **☞ Go to Question 18**

17. What motivates you to explain?

1. Due to the hospital policy
2. Because it is one of my tasks
3. Because patients want it
4. Because I receive a personal request
5. Because I have a personal interest
6. Because nobody does it
7. Other ( )

18. If you had to discuss life-sustaining treatment plans with your patients, when would it be appropriate?

1. I do not
2. When patients request
3. When an attending physician requests
4. From the time of providing hospice care
5. From the time of available diagnosis with a terminal illness
6. From the time of confirmation of imminent death
7. From the time of initial diagnosed
8. Other ( )

19. When you discuss life-sustaining treatment plans directly with patients, what do you think are the two most challenging things? 1^st^ ( ), 2^nd^ ( )

**(Nurse factor)**

1. While it is predicted to be terminal, there is no certainty if the current condition is appropriate for life-sustaining treatment decisions.
2. I am unsure of how much information I should provide about the life-sustaining treatment plans.
3. When a nurse explains, patients do not accept it well.
4. I am reluctant to explain the discontinuation of life-sustaining medical care because I feel as if I am giving up.
5. There is not enough time to discuss the discontinuation of life-sustaining treatment.
6. I am worried that I will talk differently with the doctors.
7. I am not sure if it is a nurse's job.
8. It appears as if families might be against it.
9. I do not know how to explain life-sustaining treatment decisions to the patients.
10. After explaining the discontinuation of life-sustaining medical care, I worry that patients are disappointed and frustrated.

**(Patient Factor)**

1. Patients demand treatments till the end.
2. Patients need to understand the purpose and intent of the life-sustaining treatment decision.
3. Patients do not understand terms such as “cardiopulmonary resuscitation” and “ventilator.”
4. Patients are hesitant or feel burdened to make decisions about life-sustaining medical care.
5. Patients want their doctor to decide on life-sustaining medical care.
6. Patients want their families to decide on life-sustaining medical care.

**(Family factor)**

1. Family members are against explaining life-sustaining treatment decisions to the patients.
2. Family members want to make life-sustaining treatment decisions on behalf of the patients.
3. It is challenging to coordinate opinions due to conflicts or disagreements within the family.
4. Other ( )

20. What do you think are the two most important things that are needed to facilitate discussions about life-sustaining medical decisions? 1^st^ ( ), 2^nd^ ( )

1. We need tools that can objectively assess the prognosis.
2. Documentation related to life-saving treatment decisions should be streamlined to reduce the time required for administrative procedures.
3. There needs to be compensation for the time spent discussing life-sustaining treatment decisions.
4. Training in communication should be provided so that we can better discuss the life-sustaining treatment decisions.
5. Regulations should be placed to protect medical personnel when making life-sustaining treatment decisions.
6. There is a need for materials (leaflets, videos) to explain life-sustaining treatment decisions to patients.
7. There is a need for personnel who can consult with patients and families about life-sustaining treatment decisions.
8. There is a need for a team of experts who can provide ethical and legal advice on life-sustaining treatment decisions.
9. Standardized guidelines are needed for life-sustaining treatment decisions.
10. It is necessary to maintain the existing DNR.
11. Other ( )

21. What do you think are the ***two most difficult decisions*** about the discontinuation of end-of-life treatment? 1^st^ ( ), 2^nd^ ( )

1. It is difficult for an attending physician and two fellows of related specialties to determine the end-of-life stage.
2. The process involved in decisions to discontinue end-of-life treatment is complex.
3. There are too many documents related to decisions regarding discontinuation of end-of-life treatments.
4. At the end-of-life stage, patients are unconscious, making it difficult to confirm their will to discontinue the life-sustaining treatment.
5. Getting signatures, videos, and voice records for end-of-life patients is practically difficult.
6. In many cases, getting agreements from the entire family is impossible.
7. At the end of life, family opinions are more important.
8. Other ( )

The following are questions about end-of-life care.

22. Which of the following factors make end-of-life care most difficult in inpatient clinics? 1^st^ ( ), 2^nd^ ( ), 3^rd^ ( )

1. High patient severity and understaffing, resulting in a heavy workload
2. Lack of knowledge about end-of-life symptoms
3. Lack of end-of-life care experience
4. Fear of dying
5. Absence of end-of-life care manuals
6. Poor end-of-life environment (lack of single rooms, cost of a single room, etc.)
7. The feeling of despondency after an end-of-life experience
8. Sense of not providing enough emotional support to patients, who face the end of life, and their family members
9. Caregivers not accepting death
10. Other ( )

23. What are the top ***two*** reasons patients and family members have been dissatisfied with your end-of-life care? 1^st^ ( ), 2^nd^ ( )

1. Notice of remaining lifetime by doctors
2. Lack of explanation about end-of-life and related conditions to caregivers by nurses
3. Lack of experience in end-of-life care
4. Lack of accurate knowledge of end-of-life care
5. Lack of understanding of end-of-life care
6. Complaints about procedures after the death of end-of-life patients
7. Complaints about the hospital room and environment
8. When an attending physician is late in declaring death: delay in end-of-life care
9. Other ( )
